# *In Vitro* Sensitization of Erythrocytes to Programmed Cell Death Following Baicalein Treatment

**DOI:** 10.3390/toxins6092771

**Published:** 2014-09-18

**Authors:** Rosi Bissinger, Abaid Malik, Sabina Honisch, Jamshed Warsi, Kashif Jilani, Florian Lang

**Affiliations:** 1 Department of Physiology, University of Tübingen, Gmelinstr. 5, 72076 Tuebingen, Germany; E-Mails: ro.bissinger@gmx.de (R.B.); malik.abaid@googlemail.com (A.M.); honisch.s@gmx.de (S.H.); jamshedwarsi@yahoo.com (J.W.); 2 Department of Biochemistry, University of Agriculture, 38040 Faisalabad, Pakistan; E-Mail: kashif_cbc@yahoo.com

**Keywords:** phosphatidylserine, Baicalein, Calcium, cell volume, ceramide, eryptosis

## Abstract

The polyphenolic flavonoid Baicalein has been shown to trigger suicidal death or apoptosis of tumor cells and is thus considered for the prevention and treatment of malignancy. Similar to apoptosis of nucleated cells, erythrocytes may enter eryptosis, the suicidal erythrocyte death characterized by cell shrinkage and cell membrane scrambling with phosphatidylserine translocation to the erythrocyte surface. Stimulators of eryptosis include increase of cytosolic Ca^2+^-activity ([Ca^2+^]*_i_*) and ceramide. The present study explored whether Baicalein stimulates eryptosis. To this end, forward scatter was taken for measurement of cell volume, annexin-V-binding for phosphatidylserine-exposure, Fluo3 fluorescence for [Ca^2+^]*_i_* and fluorescent antibodies for ceramide abundance. As a result, a 48 h exposure of human erythrocytes to Baicalein was followed by significant decrease of forward scatter (≥10 µM), significant increase of the percentage of annexin-V-binding cells (≥25 µM), significant increase of [Ca^2+^]*_i_* (50 µM) and significant increase of ceramide abundance (50 µM). The effect of Baicalein (50 µM) on annexin-V-binding was significantly blunted but not abrogated by removal of extracellular Ca^2+^. In conclusion, at the concentrations employed, Baicalein stimulates suicidal erythrocyte death or eryptosis, an effect at least in part due to the combined effects of Ca^2+^ entry and ceramide formation.

## 1. Introduction

Baicalein, a major polyphenolic flavonoid from dried roots of *Scutellaria baicalensis* [1], has been shown to protect against a wide variety of malignancies [2,3,4,5,6,7,8]. Baicalein is effective at least in part by triggering apoptosis [2,4,7,9,10,11,12,13,14]. On the other hand, Baicalein may protect against apoptosis [15,16,17,18]. Along those lines, Baicalein is a potent free radical scavenger and xanthine oxidase inhibitor supporting endothelial function and protecting against oxidative stress-induced cell injury [1]. Cellular mechanisms involved in the effects of Baicalein include suppression of the transcription factor NF-κB [11,19,20], modulation of the PI3K/Akt pathway [3,12,21] and mitochondria-dependent caspase activation [9].

Similar to apoptosis of nucleated cells, erythrocytes may enter eryptosis, a suicidal erythrocyte death characterized by cell shrinkage and cell membrane scrambling with translocation of phosphatidylserine to the cell surface [22]. Eryptosis is stimulated by increase of cytosolic Ca^2+^ concentration ([Ca^2+^]*_i_*), which is followed by activation of Ca^2+^-sensitive K^+^ channels with subsequent K^+^ exit, hyperpolarization, Cl^−^ exit and thus cellular loss of KCl and water with subsequent cell shrinkage [23] as well as by cell membrane scrambling with subsequent phosphatidylserine exposure at the erythrocyte surface [22]. Stimulators of eryptosis further include ceramide formation [24], caspase activation [25,26,27,28,29] and deranged activities of AMP activated kinase AMPK [30], casein kinase 1α [31,32], cGMP-dependent protein kinase [26], Janus-activated kinase JAK3 [33], protein kinase C [34], p38 kinase [35], PAK2 kinase [36], sorafenib sensitive kinases [37] and sunitinib sensitive kinases [38].

Eryptosis is elicited by a wide variety of xenobiotics [24,37,38,39,40,41,42,43,44,45,46,47,48,49,50,51,52,53,54,55,56,57,58,59,60,61,62,63,64,65,66,67,68] and is observed in several clinical conditions including sepsis, malaria, sickle cell disease, Wilson’s disease, iron deficiency, malignancy, metabolic syndrome, diabetes, renal insufficiency, hemolytic uremic syndrome, hyperphosphatemia and phosphate depletion [22,69]. However, to the best of our knowledge, experiments exploring an effect of the polyphenolic flavonoid Baicalein on eryptosis have never been reported.

The present study thus tested whether Baicalein stimulates eryptosis. To this end, human erythrocytes were incubated in Ringer with or without presence of Baicalein and cell volume, phosphatidylserine abundance at the cell surface, [Ca^2+^]*_i_*, as well as ceramide abundance determined utilizing flow cytometry.

## 2. Results and Discussion

The present study explored the influence of the polyphenolic flavonoid Baicalein on eryptosis, the suicidal erythrocyte death characterized by cell shrinkage and phosphatidylserine translocation to the cell surface.

**Figure 1:**
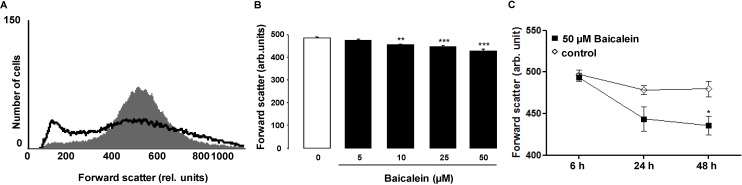
Effect of Baicalein on erythrocyte forward scatter. (**A**) Original histogram of forward scatter of erythrocytes following exposure for 48 h to Ringer solution without (grey area) and with (black line) presence of 50 µM Baicalein; (**B**) Arithmetic means ± SEM (*n* = 12) of the normalized erythrocyte forward scatter (FSC) following incubation for 48 h to Ringer solution without (white bar) or with (black bars) Baicalein (5–50 µM). ** (*p* < 0.01), *** (*p* < 0.001) indicate significant difference from the absence of Baicalein (ANOVA); (**C**) Arithmetic means ± SEM (*n* = 5) of forward scatter (arbitrary units) in erythrocytes exposed for 6–48 h to Ringer solution without (white squares) or with 50 µM Baicalein (black squares). * (*p* < 0.05) indicates significant difference from the absence of Baicalein.

In a first step, cell volume was estimated from forward scatter determined in flow cytometry following an incubation of human erythrocytes for 48 h in Ringer solution without or with Baicalein (5–50 µM). As shown in Figure 1, Baicalein treatment was followed by a decrease of average erythrocyte forward scatter reflecting cell shrinkage, an effect reaching statistical significance at 10 µM Baicalein concentration. The histogram reveals that Baicalein increases forward scatter in a subpopulation of erythrocytes.

**Figure 2:**
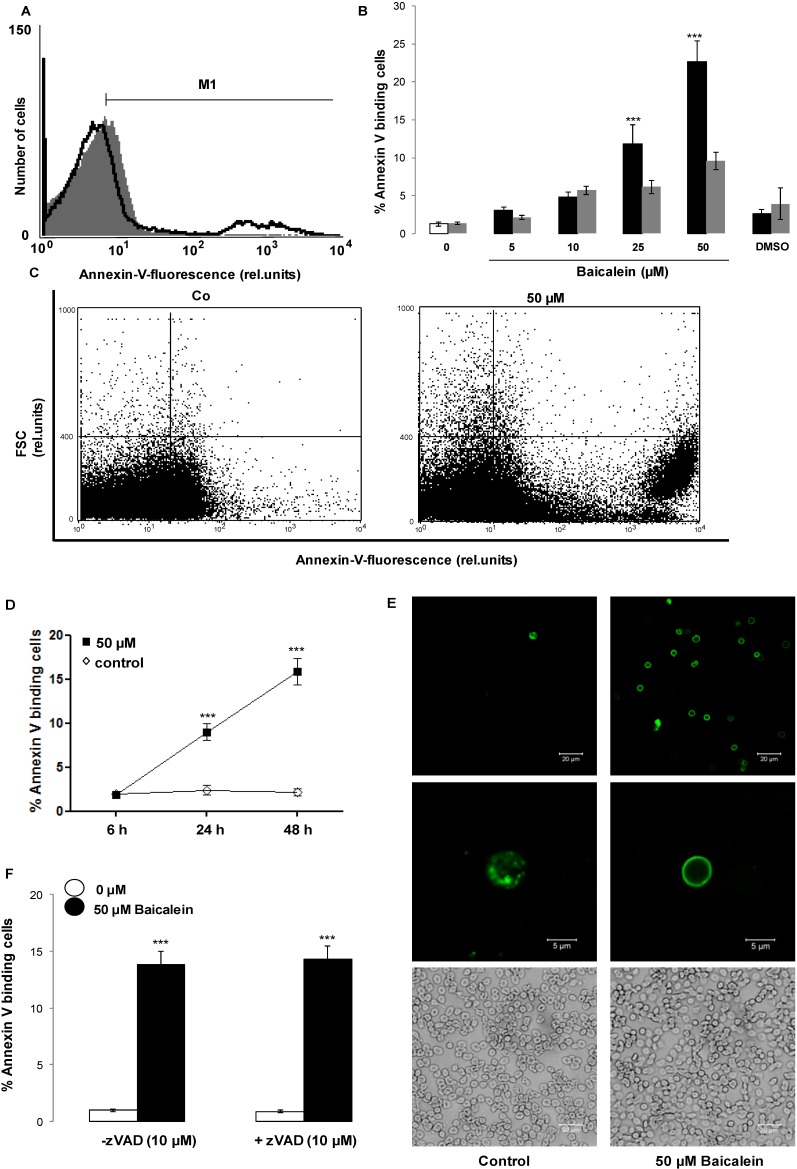
Effect of Baicalein on phosphatidylserine exposure. (**A**) Original histogram of annexin-V-binding erythrocytes following exposure for 48 h to Ringer solution without (grey area) and with (black line) presence of 50 µM Baicalein; (**B**) Arithmetic means ± SEM of erythrocyte annexin-V-binding (*n* = 12) following incubation for 48 h to Ringer solution without (white bar) or with (black bars) presence of Baicalein (5–50 µM). *** (*p* < 0.001) indicates significant difference from the absence of Baicalein (ANOVA). For comparison, the arithmetic means ± SEM of hemolysis (*n* = 4) following incubation for 48 h to Ringer solution without or with presence of Baicalein is shown (grey bars); (**C**) Original dot blots of forward scatter as a function of annexin-V-binding following exposure for 48 h to Ringer solution without and with presence of 50 µM Baicalein; (**D**) Arithmetic means ± SEM (*n* = 5) of annexin-V-binding erythrocytes (arbitrary units) following exposure for 6–48 h to Ringer solution without (white squares) or with 50 µM Baicalein (black squares). *** (*p* < 0.001) indicates significant difference from the absence of Baicalein; (**E**) Confocal images of FITC dependent fluorescence (upper panels) and light microscopy (lower panels) of human erythrocytes stained with FITC-conjugated annexin-V following a 48 h exposure to Ringer without (left panels) or with (right panels) 50 µM Baicalein; (**F**) Arithmetic means ± SEM (*n* = 5) of the percentage of annexin-V-binding erythrocytes after a 48 h treatment with Ringer solution without (white bars) or with 50 µM Baicalein (black bars) in the absence (left panels) and presence (right panels) of 10 µM pancaspase inhibitor zVAD. *** (*p* < 0.001) indicates significant difference from the absence of Baicalein (ANOVA).

In a second step, cell membrane phospholipid scrambling with phosphatidylserine translocation to the erythrocyte surface was quantified utilizing annexin-V-binding in flow cytometry following a 48 h incubation in Ringer solution without or with Baicalein (5–50 µM). As illustrated in Figure 2, a 48 h treatment with Baicalein increased the percentage of annexin-V-binding erythrocytes, an effect reaching statistical significance at 25 µM Baicalein concentration.

The effect of Baicalein on phosphatidylserine exposure is paralleled by hemolysis, which, however, affects fewer erythrocytes than cell membrane scrambling (Figure 2). The phosphatidylserine exposure was not modified by inhibition of caspases with the pancaspase inhibitor zVAD (10 µM).

**Figure 3:**
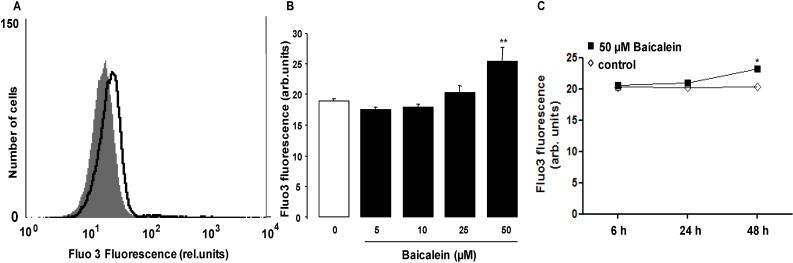
Effect of Baicalein on erythrocyte cytosolic Ca^2+^ concentration. (**A**) Original histogram of Fluo3 fluorescence in erythrocytes following exposure for 48 h to Ringer solution without (grey area) and with (black line) presence of 50 µM Baicalein; (**B**) Arithmetic means ± SEM (*n* = 12) of the Fluo3 fluorescence (arbitrary units) in erythrocytes exposed for 48 h to Ringer solution without (white bar) or with (black bars) Baicalein (5–50 µM). ** (*p* < 0.01) indicates significant difference from the absence of Baicalein (ANOVA); (**C**) Arithmetic means ± SEM (*n* = 5) of Fluo 3 fluorescence (arbitrary units) in erythrocytes exposed for 6–48 h to Ringer solution without (white squares) or with 50 µM Baicalein (black squares). * (*p* < 0.05) indicates significant difference from the absence of Baicalein.

Cell shrinkage and cell membrane scrambling with phosphatidylserine translocation to the cell surface are both known to be stimulated by increase of cytosolic Ca^2+^ activity ([Ca^2+^]*_i_*). Thus, a further series of experiments was performed to elucidate the effect of Baicalein on [Ca^2+^]*_i_*. Erythrocytes were loaded with Fluo3-AM and the Fluo3 fluorescence determined by flow cytometry following incubation for 48 h in Ringer solution without or with Baicalein (5–50 µM). As illustrated in Figure 3, exposure of the erythrocytes to Baicalein increased the Fluo3 fluorescence, an effect reaching statistical significance at 50 µM Baicalein concentration.

**Figure 4:**
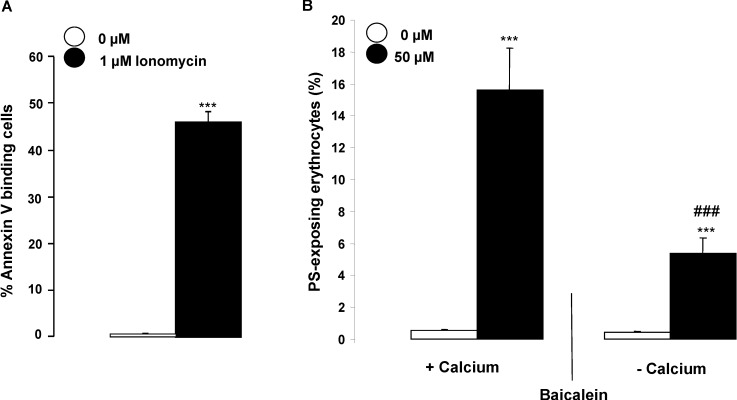
(**A**) Effect of ionomycin on phosphatidylserine exposure. Arithmetic means ± SEM (*n* = 5) of the percentage of annexin-V-binding erythrocytes following incubation for 1 h in the absence (white bar) or presence (black bar) of 1 µM ionomycin. *** (*p* < 0.001) indicates significant difference from the absence of 1 µM ionomycin (ANOVA); (**B**) Effect of Ca^2+^ withdrawal on Baicalein- induced annexin-V-binding. Arithmetic means ± SEM (*n* = 5) of the percentage of annexin-V-binding erythrocytes after a 48 h treatment with Ringer solution without (white bars) or with (black bars) 50 µM Baicalein in the presence (left bars, +Calcium) and absence (right bars, −Calcium) of calcium. *** (*p* < 0.001) indicates significant difference from the respective values in the absence of Baicalein, ### (*p* < 0.001) indicates significant difference from the respective value in the presence of Ca^2+^ (ANOVA).

Exposure of the erythrocytes to the Ca^2+^ ionophore ionomycin was followed by a strong increase of annexin-V-binding (Figure 4). In order to test, whether the Baicalein-induced cell membrane scrambling required entry of extracellular Ca^2+^, erythrocytes were exposed for 48 h to 50 µM Baicalein in the presence or nominal absence of extracellular Ca^2+^. As illustrated in Figure 4, the effect of Baicalein on annexin-V-binding was significantly blunted in the nominal absence of Ca^2+^. Nevertheless, even in the nominal absence of extracellular Ca^2+^, the percentage of annexin-V-binding erythrocytes was significantly higher in the presence than in the absence of Baicalein. Thus, Baicalein was effective partially, but not exclusively, through stimulation of Ca^2+^ entry.

**Figure 5:**
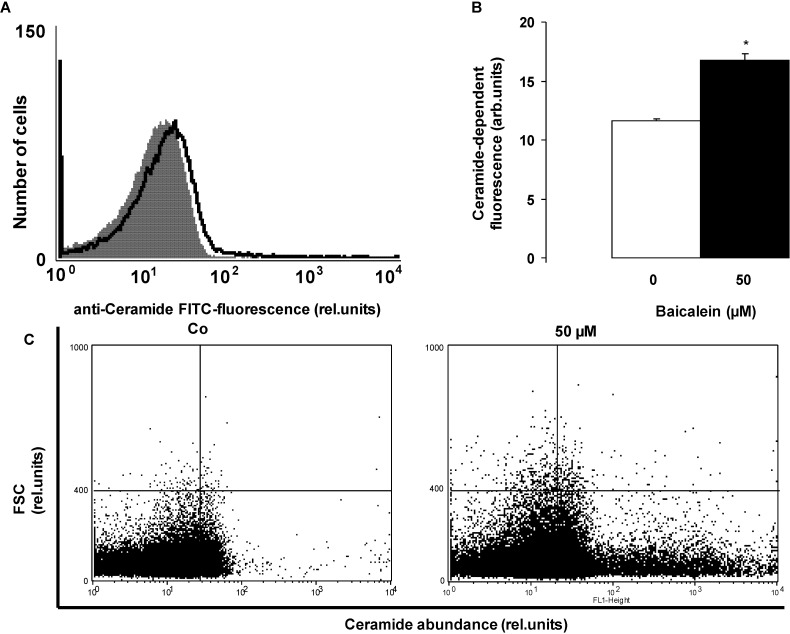
Effect of Baicalein on ceramide formation. (**A**) Original histogram of ceramide surface abundance of erythrocytes following exposure for 48 h to Ringer solution without (grey shadow) and with (black line) presence of 50 µM Baicalein; (**B**) Arithmetic means ± SEM (*n* = 5) of ceramide abundance after a 48 h incubation in Ringer solution without (white bar) or with 50 µM Baicalein (black bar). * (*p* < 0.05) indicates significant difference from the absence of Baicalein (*t*-test); (**C**) Original dot blots of forward scatter as a function of ceramide abundance following exposure for 48 h to Ringer solution without and with the presence of 50 µM Baicalein.

In search for an additional mechanism triggering eryptosis following Baicalein treatment, further experiments were performed to possibly disclose an effect of Baicalein on ceramide formation. Ceramide abundance at the erythrocyte surface was quantified utilizing an anti-ceramide antibody. As illustrated in Figure 5, exposure of erythrocytes to 50 µM Baicalein significantly increased the ceramide abundance at the erythrocyte surface.

The present study discloses a novel effect of Baicalein, *i.e.*, stimulation of eryptosis, the suicidal death of erythrocytes. Incubation of human erythrocytes with Baicalein is followed by cell membrane scrambling with phosphatidylserine translocation to the erythrocyte surface, the most important hallmark of eryptosis. The Baicalein induced cell membrane scrambling affected only a subpopulation of the erythrocytes, an observation pointing to variable vulnerability of the erythrocytes. According to an earlier study, the susceptibility against several triggers of eryptosis is enhanced in aged erythrocytes [44].

On average, Baicalein further decreases cell volume, another hallmark of eryptosis. However, Baicalein exposure increases cell volume of an erythrocyte subpopulation. This observation again points to heterogeneity among the erythrocytes, which may be similarly due to differences in erythrocyte age. The Baicalein concentrations (10–25 µM) required for those effects were in the range of the peak concentrations reported in Baicalein treated rats [70]. However, Baicalein was not immediately effective but a 24 h exposure of erythrocytes to the substance was required in order to trigger eryptosis. Whether or not those high concentrations could be maintained *in vivo* for 24 h remains uncertain.

Baicalein increased cytosolic Ca^2+^ activity ([Ca^2+^]*_i_*), an effect presumably due to stimulation of cation channels in the cell membrane. Earlier studies revealed that the erythrocyte cation channels involve TRPC6 [22].

The cell shrinkage following Baicalein treatment was presumably the result of Ca^2+^ entry with subsequent increase of [Ca^2+^]*_i_*, activation of Ca^2+^ sensitive K^+^ channels, K^+^ exit, cell membrane hyperpolarisation, Cl^−^ exit and thus cellular loss of KCl accompanied by osmotically driven water [23]. The cellular loss of KCl with water serves to counteract the swelling and subsequent hemolysis of injured erythrocytes. Hemolysis leads to release of hemoglobin, which is subject to glomerular filtration with subsequent precipitation in the acidic lumen of renal tubules [71]. The swelling of some erythrocytes following Baicalein exposure may result from Na^+^ entry through the unselective cation channel.

The stimulation of cell membrane scrambling by Baicalein is similarly in part due to increase of [Ca^2+^]*_i_*. Accordingly, the effect of Baicalein on phosphatidylserine translocation is in part dependent on entry of extracellular Ca^2+^.

However, even in the absence of extracellular Ca^2+^, Baicalein treatment is still followed by a significant increase of phosphatidylserine exposure. The residual effect is in part due to stimulation of ceramide formation. Ceramide is a well-known stimulator of eryptosis [22].

Similar to what has been shown for several other stimulators of eryptosis [22], the effect of Baicalein was not sensitive to the pancaspase inhibitor zVAD and thus did not require activation of caspases.

Consequences of excessive eryptosis include anemia, since phosphatidylserine exposing eryptotic erythrocytes are phagocytosed and thus rapidly cleared from circulating blood [22]. Anemia is prevented as long as accelerated clearance of erythrocytes during stimulated eryptosis is compensated by a similarly accelerated formation of new erythrocytes [22].

At least in theory, phosphatidylserine exposing erythrocytes may further interfere with microcirculation [72,73,74,75,76,77], as phosphatidylserine exposing erythrocytes adhere to endothelial CXCL16/SR-PSO [73], stimulate blood clotting and trigger thrombosis [72,78,79]. Baicalein has, however, been shown to counteract thrombosis and to inhibit thrombin-induced production of plasminogen activator inhibitor-1, and endothelial adhesion molecule expression [1]. Accordingly, Baicalein and its analogs have been proposed for the treatment of arteriosclerosis and hypertension [1].

Elimination of phosphatidylserine exposing erythrocytes may protect against untoward effects of hemolysis [22]. The removal of phosphatidylserine exposing erythrocytes further impacts on the clinical course of malaria [80]. Infected erythrocytes undergo eryptosis [80], since the intraerythrocytic pathogen activates ion channels including the Ca^2+^-permeable erythrocyte cation channels [81,82]. Subsequent clearance of phosphatidylserine exposing infected erythrocytes from circulating blood decreases parasitemia and by the same token precedes and thus prevents hemolysis of the parasitized erythrocytes [80]. Accordingly, the clinical course of malaria is ameliorated by genetic disorders sensitizing erythrocytes to eryptosis, such as sickle-cell trait, beta-thalassemia-trait, homozygous Hb-C and G6PD-deficiency, [22,83,84,85], by conditions with enhanced eryptosis, such as iron deficiency [86], and by eryptosis stimulating xenobiotics, such as lead [87], chlorpromazine [88] or NO synthase inhibitors [89]. In theory, Baicalein may similarly decrease parasitemia in malaria.

## 3. Experimental Section

### 3.1. Erythrocytes, Solutions and Chemicals

Leukocyte-depleted erythrocytes were kindly provided by the blood bank of the University of Tübingen. The study is approved by the ethics committee of the University of Tübingen (184/2003V). Erythrocytes were incubated *in vitro* at a hematocrit of 0.4% in Ringer solution containing (in mM) 125 NaCl, 5 KCl, 1 MgSO_4_, 32 *N*-2-hydroxyethylpiperazine-*N*-2-ethanesulfonic acid (HEPES), 5 glucose, 1 CaCl_2_; pH 7.4 at 37 °C for 48 h. Where indicated, erythrocytes were exposed to Baicalein (Enzo Life Sciences, Lörrach, Germany) at the indicated concentrations. In Ca^2+^-free Ringer solution, 1 mM CaCl_2_ was substituted by 1 mM glycol-bis(2-aminoethylether)-*N*,*N*,*N*',*N*'-tetraacetic acid (EGTA).

### 3.2. Analysis of Annexin-V-Binding and Forward Scatter

After incubation under the respective experimental condition, 50 µL cell suspension was washed in Ringer solution containing 5 mM CaCl_2_ and then stained with Annexin-V-FITC (1:200 dilution; ImmunoTools, Friesoythe, Germany) in this solution at 37 °C for 20 min under protection from light. In the following, the forward scatter (FSC) of the cells was determined, and annexin-V fluorescence intensity was measured with an excitation wavelength of 488 nm and an emission wavelength of 530 nm on a FACS Calibur (BD, Heidelberg, Germany).

### 3.3. Measurement of Intracellular Ca^2+^

After incubation, erythrocytes were washed in Ringer solution and then loaded with Fluo-3/AM (Biotium, Hayward, CA, USA) in Ringer solution containing 5 mM CaCl_2_ and 5 µM Fluo-3/AM. The cells were incubated at 37 °C for 30 min and washed twice in Ringer solution containing 5 mM CaCl_2_. The Fluo-3/AM-loaded erythrocytes were resuspended in 200 µL Ringer. Then, Ca^2+^-dependent fluorescence intensity was measured with an excitation wavelength of 488 nm and an emission wavelength of 530 nm on a FACS Calibur (BD, Heidelberg, Germany).

### 3.4. Determination of Ceramide Formation

For the determination of ceramide abundance, a monoclonal antibody-based assay was used. After incubation, cells were stained for 1 h at 37 °C with 1 µg/mL anti ceramide antibody (clone MID 15B4, Alexis, Grünberg, Germany) in PBS containing 0.1% bovine serum albumin (BSA) at a dilution of 1:5. The samples were washed twice with PBS-BSA. Subsequently, the cells were stained for 30 min with polyclonal fluorescein isothiocyanate (FITC) conjugated goat anti-mouse IgG and IgM specific antibody (Pharmingen, Hamburg, Germany) diluted 1:50 in PBS-BSA. Unbound secondary antibody was removed by repeated washing with PBS-BSA. The samples were then analyzed by flow cytometric analysis with an excitation wavelength of 488 nm and an emission wavelength of 530 nm.

### 3.5. Statistics

Data are expressed as arithmetic means ± SEM. As indicated in the figure legends, statistical analysis was made using ANOVA with Tukey’s test as post-test and *t*-test as appropriate. *n* denotes the number of different erythrocyte specimens studied. Since different erythrocyte specimens used in distinct experiments are differently susceptible to triggers of eryptosis, the same erythrocyte specimens have been used for control and experimental conditions.

## 4. Conclusions

In conclusion, the polyphenolic flavonoid Baicalein stimulates Ca^2+^ entry and ceramide formation thus leading to subsequent erythrocyte shrinkage and erythrocyte cell membrane scrambling. Accordingly, Baicalein stimulates eryptosis, the suicidal erythrocyte death. The concentrations required for those effects are 10–50 µM.
